# A study on the extracts of Cuscuta reflexa Roxb. in treatment of cyclophosphamide induced alopecia

**DOI:** 10.1186/2008-2231-22-7

**Published:** 2014-01-06

**Authors:** Satish Patel, Vikas Sharma, Nagendra S Chauhan, Vinod K Dixit

**Affiliations:** 1 Department of Pharmaceutical Sciences, Doctor Hari Singh Gour Vishwavidyalaya, Sagar 470 003, M.P, India

**Keywords:** *Cuscuta reflexa*, Chemotherapy, Alopecia, Cyclophosphamide, Hair loss

## Abstract

**Background:**

Alopecia is a dermatological disorder with psychosocial implications on patients with hair loss. Hair loss is one of the most feared side effects of chemotherapy. Plants have been widely used for hair growth promotion since ancient times in Ayurveda, Chinese and Unani systems of medicine. The effect of extracts of *Cuscuta reflexa* Roxb. in testosterone induced alopecia was reported.

**Objective:**

In the present study, the efficacies of the extracts of *Cuscuta reflexa* in promoting hair growth in cyclophosphamide-induced hair loss have been determined.

**Materials and methods:**

The study was performed by treated with petroleum ether and ethanolic extract of *Cuscuta reflexa* at the dose 250 mg/kg in male swiss albino rats. Cyclophosphamide (125 mg/kg) was used to induce alopecia.

**Results:**

Groups treated with extracts of plant showed hair regrowth. Histopathology and gross morphologic observations for hair regrowth at shaved sites revealed active follicular proliferation.

**Conclusions:**

It concluded that extracts of *Cuscuta reflexa* shown to be capable of promoting follicular proliferation or preventing hair loss in cyclophosphamide-induced hair fall.

## Introduction

Hair is a major esthetic display feature of the human body, especially in social and sexual interactions. Several drugs have an effect on hair growth and some bring about hair loss, the clinical symptoms of which are the results of interaction between the drugs and various cells important for hair growth. Such cells include follicular keratinocytes, cells of the hair matrix, peri-follicular blood vessels and those of the connective tissues surrounding the hair bulbs. The keratinocytes are a major target for the toxic effect of environmental agents or xenobiotics. This may be because up to 90% of all hair follicles are in a phase of rapid growth and the high rate of blood flow around hair bulbs results in a good bioavailability of many drugs at these sites [1].

The three major and frequent toxicities of cytotoxic cancer chemotherapy are bone marrow suppression, gastrointestinal disturbances and alopecia. Induction of massive hair loss (alopecia) by many chemotherapeutic drugs ranks among the most psychologically devastating side effects of cancer treatment. More than 80% of patients who receive chemotherapy consider hair loss the most distressing aspect of their treatment [2,3].

There have been remarkable advances in the understanding of the molecules and pathways regulating hair follicle formation, hair growth and hair loss [4,5]. Similarly, many products are now in the market for promoting hair growth and treating hair loss. However, these products often lack significant clinical efficacy in preventing the hair loss effect of chemotherapy.

*Cuscuta reflexa* Roxb. (Family Cuscutaceae), known as “amarvela” in vernacular, is a parasite, with slender yellow stems. It is widespread in temperate and tropical regions and common found throughout India. It grows on different host plants [6,7]. Traditionally, it used in treatment of protracted fever, diaphoretic, and as demulcent and as purgative [8,9]. Recent studies by Roy et al. [10,11] showed that extract and formulation of extract of *C. reflexa* Roxb. with *Citrullus colocynthis* Schrad. and *Eclipta alba* Hassk. have hair growth promoting activity and enhanced the regrowth of hair at shaved site. Similar results observed in testosterone-induced alopecia in albino mice with petroleum ether extract of *C. reflexa*[12]. In the present study, the efficacies of extracts of *C. reflexa* in counteracting cyclophosphamide (CYP) induced hair loss were investigated. The findings of this experiment might stimulate the development of a new, effective therapy for hair loss especially in patients undergoing chemotherapy.

## Materials and methods

### Plant material

Long yellow stems of *Cuscuta reflexa* growing on *Bougainvillea spectabilis* and *Jasminum multiforum* were collected in the month of Oct-Nov 2010 from forests surrounding our university campus, Sagar and were authenticated by Dr. P.K Tiwari, Department of Botany, Dr. H.S.Gour University (Herbarium no. Bot/Her/2123). The whole stem were firstly sun dried for 5–8 days and then finally dried in an oven at 35-40°C for 48 h. The dried stems were ground into coarse powders.

### Extraction

Coarsely powdered materials were extracted with petroleum ether (60–80°C) until complete extraction in soxhlet apparatus. The solvent from the extract was eliminated under reduced pressure (yield 3.0% w/w). The marc obtained after petroleum ether extraction was subjected to ethanol (95%) extraction in soxhlet apparatus for 10–16 h at 70-80°C. The solvent from the extract was eliminated under reduced pressure (yield 3.6% w/w).

### Chemicals

Cyclophosphamide (CYP) was purchased from Himedia Laboratories (Mumbai, India). Tween 80, Petroleum ether and ethanol were all from Sigma-Aldrich (India).

### Test samples

Cyclophosphamide (CYP) solution (125 mg/kg body weight) was prepared in distilled water. Petroleum ether extract suspension (250 mg/kg body weight) was prepared with help of tween-80. Ethanolic extract solution (250 mg/kg body weight) was prepared in distilled water. Distilled water use as a vehicle.

### Animal

Twenty-Four male Swiss albino rats (3-4months age, 120-140 g) were used for experiments. The animals were housed in standard cages with free access to food and water. The animal house temperature was maintained at 23 ± 1°C with a 12 hour light and12 hour dark cycle. The Institutional Ethical Committee of Dr H.S. Gour University (Reg. No. 319/01/ab/CPCSEA) approved the protocol of all animal experimentation. The guidelines of CPCSEA, India were strictly followed.

### Grouping of animals

The animals were randomly divided in 4 groups of 6 male Swiss albino rats and were treated as follows:

Group I: Vehicle only (Distilled water) (Control).

Group II: CYP solution (125 mg/Kg) (i.p.) (Negative control).

Group III: CYP solution (125 mg/Kg) (i.p.) + Petroleum ether extract solution (250 mg/Kg) of *C. reflexa* (orally).

Group IV: CYP solution (125 mg/Kg) (i.p.) + Ethanolic extract solution (250 mg/Kg) of *C. reflexa* (orally).

### Cyclophosphamide (CYP) induced Alopecia

Cyclophosphamide is one of the most often used anticancer drugs. During chemotherapy, alopecia is inducing because of follicle dystrophy and/or the premature induction of follicle regression (catagen) in growing (anagen) hair follicles. The back skins of rats were depilated of hair. This was done so that, at the start of pharmacological manipulation, all hair follicles in the depilated back skin area of all rats were in exactly the same stage of anagen development (predictable and highly synchronized anagen development can only be achieved with anagen induction by depilation, as opposed to spontaneous anagen development). As soon as anagen VI stage (after 9–10 day of depilation) reached, all the rats of test groups injected once with a high dose of CYP. Extract solutions were orally administered on 2^nd^ day after CYP administered. At different time, points after CYP injection, selected rat was sacrificed to correlate visible hair phenomena with a histological profile of follicle response and recovery (morphometry) [13].

### Anagen induction

Male Rats had gone through several postnatal hair cycles were induce to enter anagen by depilation of all hair shaft (having any cycle). It was done by applying hair remover to back skin and by peeling off this mixture after some time [14]. By this technique, all depilated hair follicles immediately start in on to transform into anagen follicles (Stage-I to Stage -VI) with their associated properties. After 9–10 days of depilation, they enter in Anagen -VI stage.

### Induction of Alopecia

As soon as early anagen-VI stage reached by induced anagen follicles, i.e. on day 9^th^ post depilation, a single intraperitoneal injection of CYP was given (125 mg/kg body weight, freshly dissolved in distilled water).

### Treatment of animals for study

The method reported by Paus was followed with slight modification. Rats in-group II, group III and group IV were administered cyclophosphamide intraperitoneally (125 mg/kg body weight) once i.e. on 9^th^ day of depilation. Animals of groups III and IV were administered orally of petroleum ether extract of *Cuscuta reflexa* and ethanolic extract of *C. reflexa* respectively from the day next to CYP administration upto 19 day of study. Animals of group I were treated with vehicle only. After 19^th^ days of study, rat from each group was selected randomly and sacrificed.

### Observation

**Figure 1:**
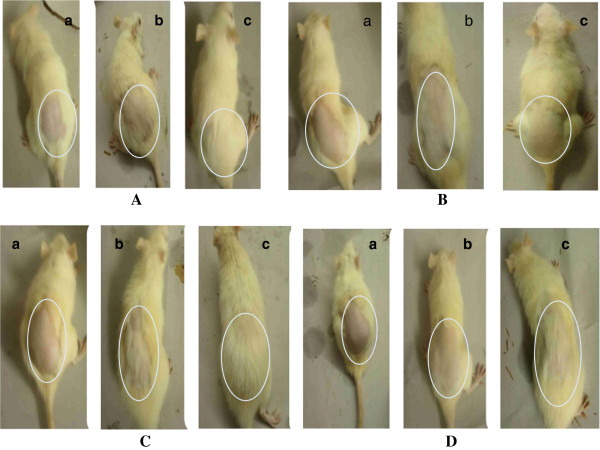
**Comparison of baldness pattern in each group. (A)** Animal treated with Vehicle only showing No hair loss **(B)** Animal treated with CYP showing diffused alopecia. **(C)** Animal treated with CYP and Pet ether extract showing less hair loss. **(D)** Animal treated with CYP and ethanolic extract showing less hair loss. a: After Depilation, b: After 9th Day of study (CYP Administered) c: After 19th Day of study.

The difference in growth of hair in each group was noticed by visual observation and was recorded by taking photographs (Figure 1A,B,C,D). Skin biopsy as well undertaken from the depilation site of each group of rat, and samples of skin were kept in phosphate-buffered formalin for paraffin sectioning. Vertical sections (3–4 μm) were cut parallel to the direction of hair growth and stained with haematoxylin and eosin. The cyclic phase of hair follicles (anagen and telogen), number of hair follicles and the anagen/telogen ratio was calculated with the help of ocular micrometer.

## Findings

### Morphologic observation

Feed and water intake did not vary significantly in Group-I animals throughout the study period. Hair re-growth at shaved sites became obvious within 2–4 days after shaving. Shaved sites were about mostly covered by day 14 and hair texture and volume compared well with unshaved areas by day 19 (Figure 1A).

In the animals of Group-II, in the first 9 days of study, they had normal feeding pattern. After CYP administration during 10-12^th^ day of study, they had normal feeding pattern but from 15^th^ day, an appreciable drop in feeding rate was observed. Hair growth at depilated site was visibly inhibited in most of the animals by day 14^th^ after an initial tiny growth. After 19^th^ day of study hair loss was significant at depilated sites in all the animals. The hairs on other parts of the animals generally had the resemblance of diffuse alopecia at day > 19 of study (Figure 1B).

In Group-III, there were no significant variations in the feeding pattern. Hair regrowth at depilated site was slow upto 14^th^ day and on 19^th^ day of study, full regrowth was observed. There was very little sign of alopecia (Figure 1C).

In animals of Group-IV, there were no significant variations in the feeding pattern. Hair regrowth at depilated site was not observed at 14^th^ day. After 14^th^ day little hair regrowth started. Hair regrowth at depilated site was visible in 80% of animal after 19th day of study. Some animal show the sign of alopecia (Figure 1D).

### Histological observation

**Figure 2:**
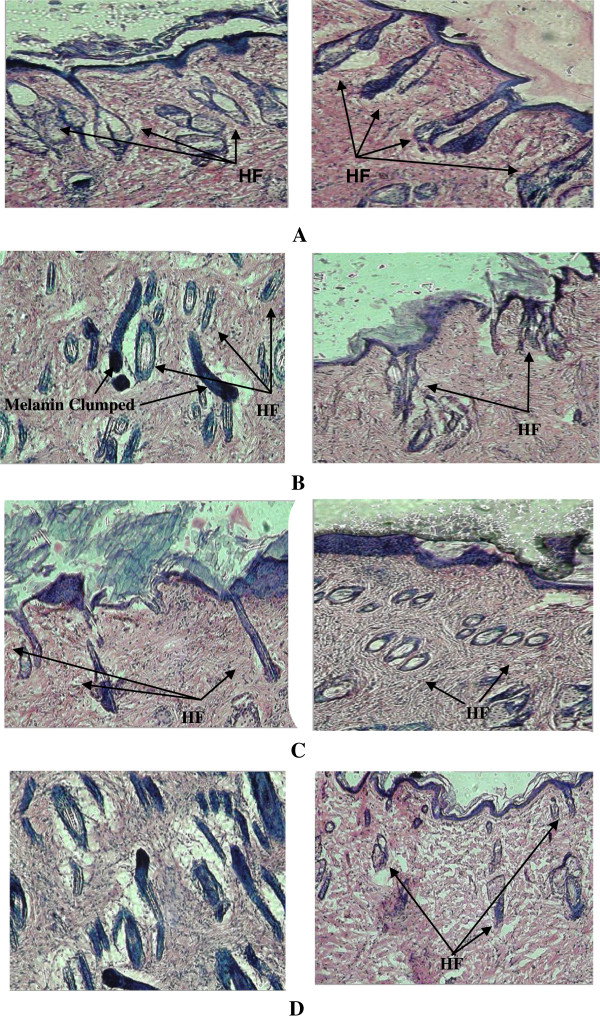
**Histology of skin sections. (A)** Skin of animal treated with Vehicle only **(B)** Skin of animal treated with CYP. **(C)** Skin of animal treated with CYP and Pet ether extract solution. **(D)** Skin of animal treated with CYP and ethanolic extract solution. (HF: Hair Follicle).

Histopathological observation in animals of group-I revealed focal areas of hypergranulosis, normal hair follicles and numerous maturing hair follicles (Figure 2A). In Group-II, skin of animal showed disruption of melanin granules, irregular banding pattern of hair shaft, disruption of epidermis, less number of hair follicles, irregular diameters of hair bulbs, wide open hair canal and distortion of hair follicles (Figure 2B). In Group-III, skin of animals showed normal epidermis, more number of hair follicles, wide open hair canal, both anagen and telogen cycle observed, melanin granules not distributed irregularly as in group-II, less distortion of hair follicle, regular diameter of hair follicles, hair shaft and fragments present (Figure 2C). Histopathologically, skin of group IV animal showed thickened and regular epidermis, less number of hair follicles, distorted hair follicles also present along with normal hair follicles, wide open hair canal, irregular diameter of hair follicle, few hair shaft fragments observed, melanin granules not distributed (Figure 2D).

### Hair density

The histological study showed that hair density was maximum *i.e.* 3.75 ± 0.62 in case of vehicle treated, 2.5 ± 0.79 in case of petroleum ether extract of *Cuscuta reflexa* treated, 2.33 ± 0.88 in case of ethanolic extract of *C. reflexa* treated, while it was minimum *i.e.* 2.08 ± 0.79 in control (CYP) treated animals.

### Anagen telogen ratio (A/T ratio)

The histological study showed that A/T ratio was maximum *i.e.* 3.20:1 in case of vehicle treated, 1.70:1 in case of petroleum ether extract of *C. reflexa* treated, 1.43:1 in case of ethanolic extract of *C. reflexa* treated, while it was minimum *i.e.* 1:2.83 in control (CYP) treated animals.

Number of anagen follicles in treated group show following order:

Group I > Group III > Group IV > Group II.

Number of telogen follicles in treated group show following order:

Group II > Group IV > Group III > Group I.

## Discussion

Cancer treatment with chemotherapeutic agents is associated with severe side effects due to the occurrence of apoptosis in several sensitive tissues (such as the hematopoietic system or epithelia of digestive tract) because of drug cytotoxicity [15]. This apoptosis largely depends on p53, a key mediator of cellular mechanism of stress response [16]. p53 accumulation in sensitive cells after a variety of stresses results in growth arrest at one of the cellular checkpoints or induction of programmed cell death [17]. Cancer chemotherapy disrupts the proliferation of matrix keratinocytes in the anagen bulb that produce the hair shaft. This induced anagen follicles to enter a dystrophic catagen stage in which the integrity of the hair shaft is compromised and the hair breaks and falls off [18]. A significant number of hair follicles are in anagen at any one time. These hairs are, however, rapidly lost during chemotherapy. CYP administration will result in enhanced deterioration of the matrix, leading to premature catagen formation and thus increased telogen shedding [19]. When administered in higher doses, CYP was found to inhibit mitosis in the hair bulb, leading to narrowing of the hair shaft, which subsequently breaks at this point. Apoptosis of hematopoietic cells and cells of the digestive tract associated with cancer treatment is recognized to be p53 dependent. Radiation- or chemotherapy-induced DNA damage leads to the rapid accumulation of p53 protein in the susceptible cells [17,20], followed by up-regulation of Fas, IGF-BP3, and Bax, encoded by the corresponding p53-responsive genes. Moreover, it was demonstrated that Fas and Bax are up regulated in the HF during cyclophosphamide treatment [4].

Many chemotherapeutic agents are strongly affecting hair follicles by because of the rapid proliferative rate of hair matrix keratinocytes during anagen. In the rat model of chemotherapy induced hair loss, the active hair growth phase was first induced by depilation, and cyclophosphamide administration during new anagen phase causes complete alopecia imitating changes seen in human chemotherapy induced hair loss. The drug treatment induces dystrophic changes in growing HF and, in more severely damaged follicles, premature regression because of massive apoptosis in the entire proximal hair bulb, with subsequent hair shedding [13,21].

The decrease in feed/water consumption especially in animals that received only CYP is due to adverse systemic effects of CYP.

Petroleum ether extract and ethanolic extract of *Cuscuta reflexa* showed the ability to prevent damage to hair follicles by CYP when administered after CYP administration and enhanced hair regrowth. Therefore, *C. reflexa* have demonstrated its ability to prevent hair loss and damage to the skin structure caused by CYP to rats in CYP induced alopecia. They possibly will be act by preventing accumulation or downregulation of p53 a key mediator of cellular mechanism of stress response. p53 accumulation in sensitive cells after a variety of stresses results in growth arrest at one of the cellular checkpoints or induction of programmed cell death. Thus preventing the apoptosis of hair follicles, dystrophic changes in growing hair follicle and preventing hair loss.

## Conclusion

In this work it was to be concluded that the petroleum ether extract and ethanolic extract of *Cuscuta reflexa* were use to prevent hair loss or to treat the alopecia (a common side effect in the cancer treatment) during chemotherapy.

## Competing interests

The authors declare that they have no competing interests.

## Authors’ contributions

SP and VKD theorized, designed and interpreted the data and drafted the manuscript. VKD, NSC and VS designed, acquainted the data and revised the manuscript. VKD theorized the Data, drafted and revised the manuscript. All authors read and approved the final manuscript.
